# Supplementary material for: The Loss-Function of KNL1 Causes Oligospermia and Asthenospermia in Mice by Affecting the Assembly and Separation of the Spindle through Flow Cytometry and Immunofluorescence
Source: Sensors (Basel). 2023 Feb 25;23(5):2571. doi: 10.3390/s23052571 (PMC10007211; doi:10.3390/s23052571)
Supplement: Supplementary file 1 [file sensors-23-02571-s001.zip › sensors-2192430-supplementary.pdf]

Supplementary Information for

The loss-function of *KNL1* causes oligospermia and asthenospermia in mice by affecting the assembly and separation of the spindle through flow cytometry and immunofluorescence

This PDF file includes Supplementary Figure S1–S3 and Table S1

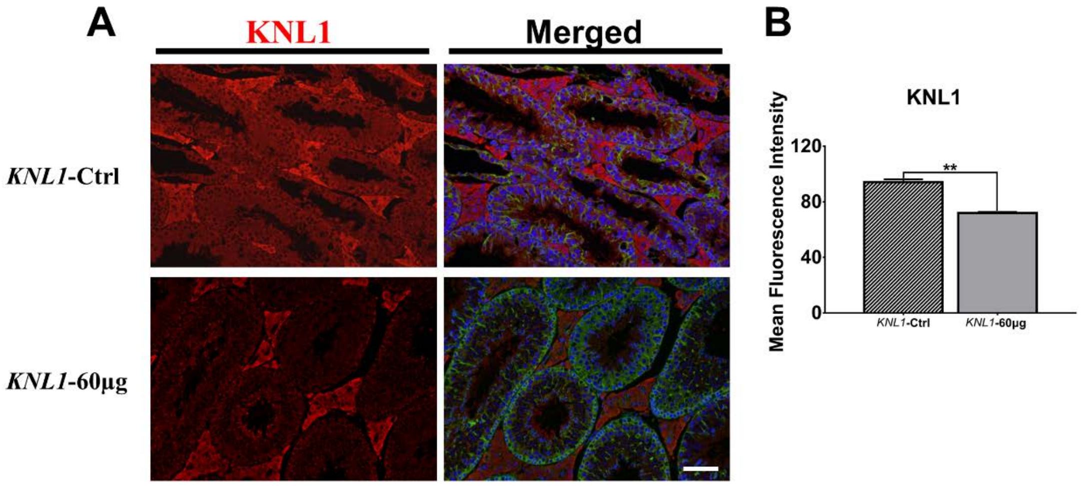

Figure S1. Immunofluorescence intensity of *KNL1* between *KNL1*-Ctrl and *KNL1*-60 μg. (**A**) *KNL1* fluorescent images of testis in *KNL1*-Ctrl and *KNL1*-60μg. Testis was immunostained with anti-*KNL1* (red). Merge was DAPI (blue), *KNL1* (red), and α-tubulin (green). (**B**) Data were presented as mean percentages (mean ± SEM) of at least three independent measurements. Asterisk denotes statistical difference level of significance (**, p < 0.01, ns > 0.05). Bar= 100 μm.

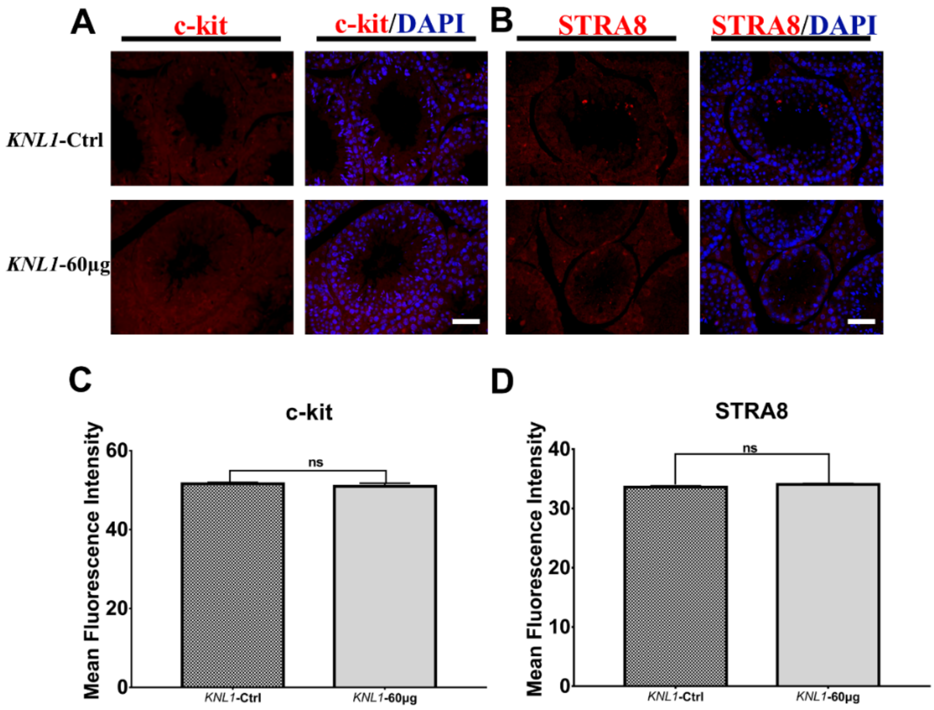

Figure S2. Immunofluorescence intensity of two markers between *KNL1*-Ctrl and *KNL1*-60 μg. (**A**)c-kit fluorescent images of testis in *KNL1*-Ctrl and *KNL1*-60 μg. Testis was immunostained with anti-c-kit(red). Merge was DAPI (blue) and c-kit (red). (**B**) STRA8 fluorescent images of testis in *KNL1*-Ctrl and *KNL1*-60 μg. Testis was immunostained with anti-STRA8 (red). Merge was DAPI (blue) and STRA8 (red). (**C**,**D**) Data were presented as mean percentages (mean ± SEM) of at least three independent measurements. ns denotes statistical difference at a $p > 0.05$ level of significance. Bar = 50 μm.

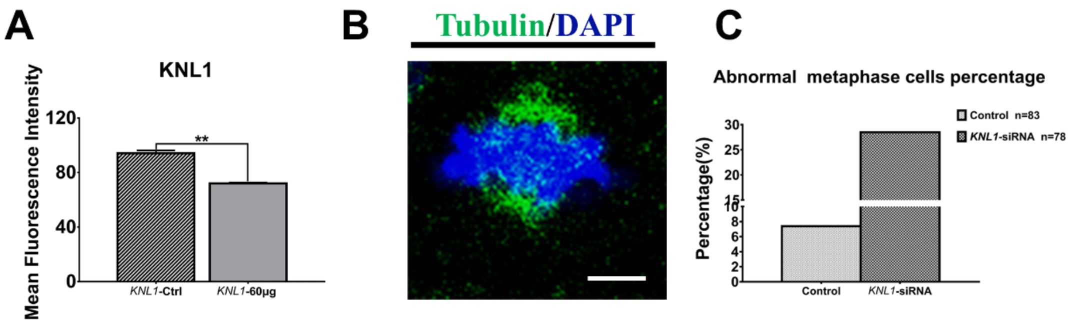

Figure S3. *KNL1* siRNA interference efficiency and immunofluorescence image of normal cell division. (**A**) *KNL1* siRNA interference efficiency in GC-2 cells. (**B**) The normal spindle and chromosome arrangement at metaphase. Bar= 5 μm. (**C**) The percentage of abnormal metaphase cells between Control-cells and *KNL1*-siRNA-cells. Asterisk denotes statistical difference level of significance (**, p < 0.01, ns > 0.05).

Table S1. Immunofluorescence antibody used in the experiment

Name	Brand
PLZF **Rabbit Polyclonal Antibody**	**Beyotime**
**Histone** ***H2AX*** **Rabbit Polyclonal Antibody**	**Beyotime**
STRA8 **Monoclonal antibody**	**Proteintech**
SYCP3 **Polyclonal antibody**	**Proteintech**
GFRα1 **Polyclonal Antibody**	**Elabscience**
**Purified Anti-Mouse c-kit Antibody**	**Elabscience**
**Alpha Tubulin Polyclonal antibody**<br>**CASC5 (E4A5L) Rabbit mAb**	**Proteintech**<br>**CST**
**Goat Anti-Rabbit IgG H&L (Alexa Fluor 594)**	**GeneCopoeia**
**CoraLite594 – conjugated Goat Anti-Mouse IgG(H+L)**	**Proteintech**
**CoraLite488 – conjugated Affinipure Goat Anti-Rabbit IgG(H+L)**	**Proteintech**
